# MetfOrmin BenefIts Lower Extremities with Intermittent Claudication (MOBILE IC): randomized clinical trial protocol

**DOI:** 10.1186/s12872-023-03047-8

**Published:** 2023-01-21

**Authors:** Katherine M. Reitz, Andrew D. Althouse, Daniel E. Forman, Brian S. Zuckerbraun, Yoram Vodovotz, Ruben Zamora, Robert L. Raffai, Daniel E. Hall, Edith Tzeng

**Affiliations:** 1 Department of Surgery, University of Pittsburgh, South Tower, Rm 351.6, 200 Lothrop Street, Pittsburgh, PA 15213 USA; 2 Division of Vascular Surgery, University of Pittsburgh, Pittsburgh, PA USA; 3 Department of Surgery, Veterans Affairs Pittsburgh Healthcare System, Pittsburgh, PA USA; 4 School of Medicine, University of Pittsburgh, Pittsburgh, PA USA; 5 Department of Medicine, University of Pittsburgh, Pittsburgh, PA USA; 6 Geriatrics Research, Education, and Clinical Care, Veterans Affairs Pittsburgh Healthcare System, Pittsburgh, PA USA; 7 Center for Inflammation and Regeneration Modeling, McGowan Institute for Regenerative Medicine, Pittsburgh, PA USA; 8 Center for Systems Immunology, University of Pittsburgh, Pittsburgh, PA USA; 9 Wolff Center, UPMC, Pittsburgh, PA USA; 10 Center for Health Equity Research and Promotion, Veterans Affairs Pittsburgh Healthcare System, Pittsburgh, PA USA; 11 VA Medical Center, San Francisco, CA USA

**Keywords:** Peripheral artery disease, Intermittent claudication, Metformin, Anti-inflammatory agents, Clinical trial protocol, Atherosclerosis

## Abstract

**Background:**

Peripheral artery disease (PAD) affects over 230 million people worldwide and is due to systemic atherosclerosis with etiology linked to chronic inflammation, hypertension, and smoking status. PAD is associated with walking impairment and mobility loss as well as a high prevalence of coronary and cerebrovascular disease. Intermittent claudication (IC) is the classic presenting symptom for PAD, although many patients are asymptomatic or have atypical presentations. Few effective medical therapies are available, while surgical and exercise therapies lack durability. Metformin, the most frequently prescribed oral medication for Type 2 diabetes, has salient anti-inflammatory and promitochondrial properties. We hypothesize that metformin will improve function, retard the progression of PAD, and improve systemic inflammation and mitochondrial function in non-diabetic patients with IC.

**Methods:**

200 non-diabetic Veterans with IC will be randomized 1:1 to 180-day treatment with metformin extended release (1000 mg/day) or placebo to evaluate the effect of metformin on functional status, PAD progression, cardiovascular disease events, and systemic inflammation. The primary outcome is 180-day maximum walking distance on the 6-min walk test (6MWT). Secondary outcomes include additional assessments of functional status (cardiopulmonary exercise testing, grip strength, Walking Impairment Questionnaires), health related quality of life (SF-36, VascuQoL), macro- and micro-vascular assessment of lower extremity blood flow (ankle brachial indices, pulse volume recording, EndoPAT), cardiovascular events (amputations, interventions, major adverse cardiac events, all-cause mortality), and measures of systemic inflammation. All outcomes will be assessed at baseline, 90 and 180 days of study drug exposure, and 180 days following cessation of study drug. We will evaluate the primary outcome with linear mixed-effects model analysis with covariate adjustment for baseline 6MWT, age, baseline ankle brachial indices, and smoking status following an intention to treat protocol.

**Discussion:**

MOBILE IC is uniquely suited to evaluate the use of metformin to improve both systematic inflammatory responses, cellular energetics, and functional outcomes in patients with PAD and IC.

*Trial Registration*: The prospective MOBILE IC trial was publicly registered (NCT05132439) November 24, 2021.

## Background

Peripheral artery disease (PAD) results from systemic atherosclerosis and is associated with chronic systemic inflammation [1] and impaired mitochondrial function [2]. It affects over 230 million patients world-wide [3]. PAD is prevalent among United States Veterans with higher rates of revascularization within the Veterans Health Administration when compared to private sector hospitalizations overall and especially at an earlier age [4–6]. While individuals with PAD may remain asymptomatic, the classic presentation for PAD is intermittent claudication (IC) [7], defined as reproducible lower extremity muscular pain with ambulation due to arterial blood flow limitations. PAD and IC are associated with an increased risk of cardiovascular disease (CVD) morbidity and mortality in addition to a progressive decline in walking distance, functional independence, and quality of life [8–10].

Treatment of IC has two goals. First, reduce CVD morbidity and mortality. Second, improve walking ability and quality of life [11, 12]. The former is achieved with optimal medical therapy (OMT) including smoking cessation, blood pressure control, as well as lipid-lowering and antiplatelet therapy. The latter is managed with cilostazol, exercise programs, and surgical revascularization [11]. Unfortunately, pharmacologic treatment of IC with cilostazol is minimally effective and poorly tolerated [12]. Exercise and revascularization can improve symptoms [13] but the improvements are not consistently sustained, are highly dependent on patient compliance, and do not reduce associated CVD morbidity and mortality [14, 15]. Further, vascular interventions to alleviate IC may lead to accelerated PAD progression to critical limb threatening ischemia [16–18].

Metformin is the most frequently prescribed oral therapy for Type 2 diabetes and has an excellent safety profile [19]. The pleiotropic effects of metformin include reducing reactive oxygen species production [20], attenuating systemic inflammation [21], and inhibiting mitochondrial damage [22, 23], all of which can improve age-related organ dysfunction [24]. Mechanistically, metformin activates AMP-activated protein kinase (AMPK) [25], increases endothelial nitric oxide synthase (eNOS) activity [26], and promotes mitochondrial biogenesis, mitophagy, and autophagy [27]. In patients with diabetes, metformin is linked to improved cellular respiration and both decreased the incidence and progression of age-related comorbidity (i.e., cancer, CVD, kidney diseases, etc.), frailty, response to physiologic stress, and mortality [24, 28, 29]. These effects appear to be independent of glucose control [24].

In patients with PAD, preclinical studies have shown that metformin stimulates angiogenesis [30] and reduces inflammatory arterial calcification [31]. Over 30 years ago in Italy, Sirtori et al. hypothesized that metformin would improve symptomatic IC and generated supportive preliminary evidence with two small clinical trials [32, 33]. We will extend this work and hypothesize that metformin will improve the functional status of non-diabetic patients with IC by preventing PAD progression and age-related CVD comorbidities through the reduction of *systemic* inflammation and improvement in *systemic* cellular respiration as would be predicted by the known effects of metformin on AMPK, eNOS, angiogenesis and mitochondrial health. We will conduct a triple-blind, Phase III, single institution, randomized controlled trial allocating participants to metformin or placebo, MetfOrmin BenefIts Lower Extremities with Intermittent Claudication (MOBILE IC) Trial (NCT05132439), to address this hypothesis.

## Methods/design

Over a 4-year study, non-diabetic Veterans with IC will be randomized 1:1 to 180-day treatment with either metformin or placebo to evaluate the effect of metformin on functional status, PAD progression, CVD events, and systemic inflammation at the Veterans Affairs Pittsburgh Healthcare System (VAPHS). The MOBILE IC trial protocol was reviewed by the Food and Drug Administration (FDA) and the VAPHS Institutional Review Board. The protocol follows Standard Protocol Items: Recommendations For Interventional Trials (SPIRIT) and meets all requirements for exception from investigational new drug application (IND145416), received regulatory approval (1622906), and has been publicly registered (ClinicalTrials.gov: NCT05132439). This clinical trial is funded by the VA Office of Research and Development’s Clinical Science Research and Development Merit Award (I01 CX002150).

### Study aims and outcomes

The MOBILE IC trial aims to establish the effectiveness of metformin on improving overall functional status, PAD progression, and systemic inflammation and cellular respiration in Veterans with PAD and IC. The primary objective is to evaluate the effectiveness of metformin as a pharmacologic treatment for PAD and IC with a Phase III randomized controlled trial. Outcomes are validated and reproducible measures will be used in the evaluation of PAD and IC.

The primary outcome of interest is the maximum walking distance (MWD) on the 6-min walk test (6MWT) [34]. This is a validated measure of functional status in PAD and IC, is highly reproducible, and correlates best with real-life walking capacity [34–36]. Secondary assessments of functional status include distance to claudication pain and rest on the 6MWT, aerobic and anaerobic capacity during cardiopulmonary exercise testing (CPET), Walking Impairment Questionnaire [37], and grip strength. CPET indices include peak oxygen uptake (VO_2_), ratio of minute ventilation (VE) to exhaled carbon dioxide (VCO_2_) or breathing efficiency (VE/VCO_2_), respiratory exchange ratio (VCO_2_/VO_2_), heart rate, and blood pressure [38, 39]. Ventilatory anaerobic threshold (i.e. change from aerobic to anaerobic metabolism, and point of IC onset during CPET are also assessed [38]. CPET measures of symptom-limited (maximal) aerobic and anaerobic capacity in patients with PAD and IC correlate with *systemic* disease severity and outcomes [38, 39]. Functional outcomes will be supported by the general (SF-36) and disease specific (Vascular Quality of Life Questionnaire-6 [VascuQoL-6]) health related quality of life questionnaires.

The MOBILE IC trial will also assess subclinical and clinical PAD outcomes. Subclinical outcomes will be assessed with ankle-brachial index (ABI), pulse volume recording (PVR), and EndoPAT®. The ABI assesses *regional* lower extremity blood supply in large conduit arteries as well as the contribution of collateral blood vessels. Because of the frequent insensitivity of ABI due to arterial calcification, we will also assess PVR to capture changes in distal blood flow. *Systemic* endothelial cell and vasomotor function will be evaluated by EndoPAT which measures peripheral artery tonometry before and during reactive hyperemia induced by temporary brachial artery occlusion [40]. The EndoPAT software (Itamar Medical, Israel) calculates both reactive hyperemia index, a measure of endothelial function, as well as an augmentation index, a measure of arterial stiffness [41]. Clinical PAD outcomes include minor and major amputations, revascularization procedures, major adverse cardiac events (MACE; i.e., composite of CVD mortality, myocardial ischemia, coronary revascularization, arrhythmia, heart failure, non-fatal stroke, and transient ischemic attack), and all-cause mortality.

These outcomes will be supported by key evaluations to further understand the biologic mechanism of action for metformin in PAD through measurements of systemic inflammatory biomarkers and mitochondrial function. Inflammatory biomarkers of interest include IL-1β, IL-10, IL-2, IL-6, MCP-1, HMGB1, and VCAM-1. We will perform exploratory studies on the effect of metformin on neutrophil extracellular traps and plasma exosome/microRNA that have been linked to systemic inflammation and atherogenesis [42, add reference for exosomes]. Mitochondrial function including basal and maximal mitochondrial function, ATP production, and coupling efficiency will be assessed in peripheral blood mononuclear cells to examine the systemic effects of metformin on cellular energetics that parallel changes in skeletal muscle [43, 44]. Measures of systemic oxygen consumption with CPET will complement these analyses.

### Study design

**Fig. 1:**
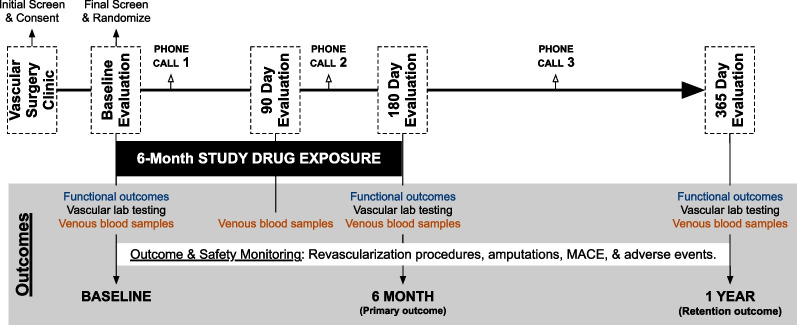
MOBILE IC trial protocol. *MACE* Major Adverse Cardiovascular Events

The MOBILE IC Trial is a single-center, triple blinded (i.e., Veteran, research staff, investigator), placebo-controlled trial testing the effectiveness of 180 days of two over-encapsulated metformin extended release (ER) tablets (500 mg each, 1000 mg/day) versus two placebo capsules daily for non-diabetic Veterans with PAD and IC. The research activities of the MOBILE IC trial, guided by a qualitative survey of Veteran interest in trial participation and visit frequency, are summarized in Fig. 1. In preparation for this trial, a checklist reviewing OMT among all patients evaluated in VAPHS vascular surgery clinic was implemented in September of 2019.

A centralized data monitoring committee (DMC) has been assigned by the VA Clinical Science Research and Development (CSRD) to ensure independent oversight of the safety and integrity of the trial without conflict of interest. The responsibilites of the DMC are documented in a Charter that has been approved by the PI, the DMC Chairperson, and the Director of CSRD. The DMC will review study enrollment, adverse events, unexpected problems, unblinded study data, and recruitment as well as retention every 12 months. Due to the excellent safety profile and long history of clinical Metformin use, no interim analysis will be completed.

#### Randomization, allocation concealment, and blinding

Study participants will be recruited from both in-person and virtual VAPHS vascular surgery clinics. Individuals meeting all inclusion and no exclusion criteria and agree to participate will be randomly assigned 1:1 to 180 days of metformin or placebo treatment (Table 1). The randomization table has been generated by the study statistician and provided to VAPHS Investigational Drug Service (IDS) for study drug allocation. The IDS will maintain the list of allocations for the duration of the study, but has no role in study recruitment, thereby preserving allocation concealment at the point of enrollment. The research staff enrolling participants have no knowledge of allocations. When an eligible participant is enrolled in the study, the VAPHS IDS is informed who then selects the next allocation from the correct sequence. Randomization will be stratified on baseline MWD on the 6MWT as guided by Fontaine Classification (Stage IIA: IC pain at ≥ 200 m; Stage IIB: < 200 m) [45] and smoking status. Within each stratum, variable block sizes (ranging from 2 to 6) are used to further protect allocation concealment. As a placebo-blinded trial, participants and research staff will not know their assignment, but variable block sizes within each stratum provides an additional layer of protection against research staff guessing future allocations. Stratified randomization is employed to maximize statistical power and allow for non-biased subgroup analysis [46].

**Table 1:** Inclusion and exclusion criteria

Inclusion criteria: subjects must meet all the following	
	Male and female Veterans > 35 and < 89 years of age
	Symptoms of intermittent claudication
	Diagnosis of PAD as defined by ABI < 0.9 or $\ge$ 0.9 with evidence of PAD as documented by pulse volume recordings (within 180 days prior to expected randomization)
	Medically stable, optimal medical therapy ($>$ 3 months prior to randomization)
	Maximum Walking Distance (MWD) on the 6-min walk test (6MWT) of ≥ 50 m with onset of pain before or at 400 m without the use of a walker
Exclusion criteria*:* subjects cannot possess any of the following	
General	
	Investigator expects inclusion could cause harm to subject
	Currently taking metformin or have taken metformin (within 180 days of expected randomization date)
	Medical condition that will limit ability to ambulate other than PAD or life expectancy (angina, congestive heart failure, pulmonary disease requiring continuously supplemented oxygen, malignancy requiring treatment, etc.)
Pre-existing diabetes	
	Type I or II Diabetes Mellitus
	HemoglobinA1c > 6.5 (within 180 days of expected randomization date)
Claudication	
	Prior above or below knee amputation
	Planned hospital admission, major operation, or lower extremity revascularization (< within 12 months after expected randomization date)
	Critical limb threatening ischemia (define by non-healing wounds and/or rest pain)
	Prior major operation or lower extremity revascularization (within the 3 months before expected randomization date)
HRQoL testing participation	
	Non-English speaking
	Dementia
Metformin safety	
	Kidney disease (dialysis dependence and/or estimated glomerular filtration rate < 45 within 180 days of expected randomization date)
	Planned iodinated contrasted imaging study (within the 3 months of expected randomization date)
	Acute or chronic metabolic acidosis with or without coma
	Women who are pregnant or breast feeding
	Unable to swallow uncrushed study drug pills
	Enrollment in another greater than minimal risk study

#### Follow up schedule and procedures

Veterans will return at 90 $\pm \hspace{0.17em}$21, 180$\pm \hspace{0.17em}$21, and 365$\pm \hspace{0.17em}$21 days for in-person evaluation. At the baseline, 180 day and 365 day in-person encounters, Veterans will undergo the same series of sequential testing: EndoPAT, venous blood sampling, 6MWT, ABI and PVR, health related quality of life testing and walking impairment questionnaire, grip strength, followed by CPET. The 90-day visit will be used to monitor medication compliance, dispense the next 90-day supply of medication, and collect blood with sample biobanking. Clinical PAD outcomes will be monitored throughout the 12-months following study drug initiation. The 365-day evaluation is intended to examine the durability of limited exposure to study drug and further understand the causality of treatments through temporal changes.

### Study drug

Metformin is inexpensive, safe, and well tolerated in both diabetic and non-diabetic patients [47–49]. Metformin ER tablets will be over-encapsulated to match placebo capsules. In our experience and as previously published [47, 48, 50], approximately 25% of non-diabetic individuals may experience self-limited gastrointestinal symptoms (i.e., diarrhea, flatus, nausea, abdominal pain) when exposed to study drug. Veterans unable to tolerate these symptoms at any point during drug exposure will reduce dosage to 1 tablet daily. If symptoms persist and remain intolerable, study drug will be discontinued. If 1 tablet is tolerated for 7 days, the dose will be increased back to 2 capsules daily and maintained if tolerated [47]. Those who tolerate 1 tablet but not 2 will continue 1 tablet for the remainder of the planned exposure period.

The estimated glomerular filtration rate (eGFR) will be monitored throughout study drug exposure and as clinically indicated. Study drug will be stopped if eGFR drops to < 30. If organ dysfunction is independent of study drug, it will be re-initiated upon return of function (i.e., eGFR $\ge \hspace{0.17em}$45).

Medication tolerance and dose adjustment will be adjudicated with guidance from the DMC which will function as the Data Safety Monitoring Board. The study team will monitor study drug compliance through phone interview and with return of pill bottles at 90- and 180-day encounters. Unused study drug will be counted and then disposed. To encourage compliance and to respect the Veterans’ time commitment, monetary reimbursement will be provided for all in-person visits.

### Statistical analysis plan

#### Power analysis

In accordance with trial design and reporting guidelines, sample size calculations focused on the primary outcome and efficacy of metformin in the treatment of IC [51–53]. The sample size was calculated to provide adequate power to assess clinically meaningful differences in the primary outcome between treatment and control groups. Based on the correlation with an associated decrease in mortality and improvement in health related quality of life, a large meaningful change of MWD is defined as 20–50 m which is the equivalent of a 5–100% change over baseline [54, 55]. Supervised exercise and endovascular intervention trials in PAD found a significant increase in MWD on 6MWT compared to control over 12 to 52 weeks with the effect size $\pm \hspace{0.17em}$standard deviation range of 22.2 $\pm \hspace{0.17em}$52.1 to 53.5 $\pm \hspace{0.17em}$105.0 m [56, 57]. The aforementioned initial Italian studies of non-diabetic patients with IC found a 53% increase in exercise capacity after 180 days of metformin exposure [33].

Assuming a baseline mean MWD of 300 $\pm \hspace{0.17em}$100 m in the overall trial population with a mean improvement of 30 $\pm \hspace{0.17em}$60 m in the metformin group versus 0 $\pm \hspace{0.17em}$60 m in the placebo group, a sample size of 80 patients per group will have 85% power to detect an improvement in MWD at 180 days in response to metformin versus placebo. Stratification of baseline MWD potentially reduces the variation between randomized groups and increases statistical power [46, 58]. The magnitude of power gained cannot be precisely quantified; thus, we did not adjust our sample size estimation for stratification. Therefore, allowing for a conservative 20% dropout rate, consistent with recently completed PAD and VA trials, we will use a total sample size of 200 with 100 per treatment group [14, 59]. Study advertisement and support will be provided to the primary care and cardiology departments within the VAPHS as needed to support enrollment.

#### Outcome evaluation

The primary analysis will be completed on an intention-to-treat plan with secondary analysis completed on per-protocol study drug allocation. All analysis will be subject to an $\alpha$ level of 0.05 on two-sided testing, and findings will be reported using CONSORT guidelines [53]. Trial results will be disseminated through ClinicalTrials.gov and peer reviewed publication.

The primary outcome will be analyzed using linear mixed-effects model analysis with MWD at 180 days as the outcome variable, a fixed effect for treatment assignment, and covariate adjustment for patient baseline MWD [60, 61]. This approach addresses the study question systematically: For 2 patients with the same pre-trial MWD, one given metformin and one placebo, what is the estimated difference in MWD after 180 days of study drug exposure? The approach, with a covariate adjustment for the baseline measure, is preferred over change scores due to favorable estimation properties and increased statistical power [61]. The model will adjust for baseline covariates with known strong associations to PAD outcomes (age, smoking status, and ABI [8]) to improve precision and increase statistical power [60, 61]. As randomization will be stratified by baseline MWD (MWD < 200 or  $\ge$ 200 m) and smoking status, we will test the interaction between these factors and treatment assignment to determine if the treatment effect is different for patients across the baseline MWD stratum.

Secondary outcome analysis will mirror that of the primary outcome for continuous variables. Each of the exploratory secondary outcomes, best summarized as time-to-event (amputations, vascular interventions, MACE, and all-cause mortality), will be reported using Kaplan–Meier analysis, log-rank tests, Cox proportional-hazards, and Fine-Gray models to estimate the difference between treatment groups, controlling for the competing risk of mortality, as appropriate. The MOBILE IC trial is not powered to test for differences in these outcomes but they are included to assess safety, mechanisms of action, allow for future secondary analysis with related studies, as described below, and inform future clinical trials of metformin in PAD and other diseases.

Secondary analysis of outcomes will include per protocol analysis as well as a Bayesian statistical analysis plan.

#### Missing data

All clinical outcomes will be de-identified, collected and managed using REDCap (Research Electronic Data Capture) tools hosted at the Veterans Affairs Information Resource Center (ViREC) [62, 63]. REDCap is a secure, web-based software platform designed to support data capture for research studies, providing (1) an intuitive interface for validated data capture; (2) audit trails for tracking data manipulation and export procedures; (3) automated export procedures for seamless data downloads to common statistical packages; and (4) procedures for data integration and interoperability with external sources.

We will report all adverse events and reasons for study drop-out using a withdrawal/termination form to assess the missing data mechanism: missing completely at random, missing at random, or non-ignorable missingness. We will conduct sensitivity analyses for primary and secondary outcomes using several validated methods: (1) complete case analyses which assumes missing completely at random; (2) multiple imputation which assumes missing at random; (3) assigning poor scores for missing values differentially by treatment group which aligns with non-ignorable missingness, and (4) the composite approach proposed by Colantuoni et al. [64]. Sensitivity analyses are recommended for trials with missing data, and we will use similar methods to those used successfully in a recent VA trial [59].

### Trial harmonization and secondary analysis

The study drug exposure duration, evaluation of the primary outcome, and quality of life testing in MOBILE IC were synchronized with the Improved PAD PERformance with METformin (PERMET) trial (NCT03054519) [34–36]. The actively enrolling PERMET trial will evaluate if up to 2000 mg metformin daily for 180 days will improve 6MWT performance among individuals with PAD compared to placebo. Data from both trials will be synchronized (expected n = 412) and will allow for secondary analyses including the (1) dose–response relationship, (2) effects of metformin among pre-specified subgroups, and (3) effects of metformin on CVD events. Pre-specified subgroups include stratification by baseline age, MWD on 6MWT, ABI, and smoking status.

## Discussion

PAD is a disease of systemic inflammation and atherosclerosis often presenting as IC. Patients with PAD and IC reduce their physical activity to limit the pain associated with ambulation. The systemic nature of atherosclerosis in PAD increases their overall risk of CVD. Currently, treatments are limited and, in the United States, the FDA has not approved an effective medication for IC in over 2 decades [65]. Existing therapies include preoperative medical optimization that target systemic CVD risk factors. Lower extremity revascularization, especially endovascular techniques, is increasingly prescribed and provides some symptomatic relief. However, it does not address the underlying systemic atherosclerosis or the risk of associated CVD. Lower extremity revascularization may also accelerate the progression to critical limb threatening ischemia [16–18, 66]. Metformin has salient properties beyond that of glucose control with mounting evidence for its pro-mitochondrial and anti-inflammatory properties, potential to promote arteriogenesis, reduction in the incidence of diseases of aging including CVDs, and improved longevity [21, 24, 30]. Therefore, the evidence supports investigating the ability of metformin to improve cellular energetics, reduce systemic inflammation, and improve functional status in patients with PAD and IC. The MOBILE IC Trial will provide empiric evidence and mechanistic data for the potential effectiveness of metformin as an innovative therapy in Veterans with PAD. The a priori planned synchronization of the MOBILE IC and PERMET trials will allow for further investigation of the effects of metformin on PAD among pre-specified subgroups and in the analysis of outcomes that each trial alone is under-powered to evaluate.

## Data Availability

Data access will be limited to investigators and study personnel. The data and study materials will not be made available to other researchers because they contain subject identifiers.

## Abbreviations

PAD: Peripheral artery disease
IC: Intermittent claudication
CVD: Cardiovascular disease
OMT: Optimal medical therapy
AMPK: AMP-activated protein kinase
eNOS: Endothelial nitric oxide synthase
MOBILE IC: MetfOrmin BenefIts Lower Extremities with Intermittent Claudication
VAPHS: Veterans Affairs Pittsburgh Healthcare System
FDA: Food and Drug Administration
SPIRIT: Standard Protocol Items: Recommendations for Interventional Trials
IND: Investigational new drug application
MWD: Maximum walking distance
6MWT: 6-Minute walk test
CPET: Cardiopulmonary exercise testing
VO_2_: Peak oxygen uptake
VE: Ratio of minute ventilation
VCO_2_: Carbon dioxide
VE/VCO_2_: Breathing efficiency
VCO_2_/VO_2_: Respiratory exchange ratio
VascuQoL-6: Vascular Quality of Life Questionnaire-6
ABI: Ankle-brachial index
PVR: Pulse volume recording
ER: Extended release
IDS: Investigational Drug Service
eGFR: Estimated glomerular filtration rate
MACE: Major adverse cardiac events
REDCap: Research Electronic Data Capture
ViREC: Veterans Affairs Information Resource Center
PERMET: PAD PERformance with METformin
CSRD: Clinical Science Research and Development
DMC: Data Monitoring Committee
